# Vitamin D in Multiple Sclerosis—Lessons From Animal Studies

**DOI:** 10.3389/fneur.2021.757795

**Published:** 2021-10-20

**Authors:** Michaela Tanja Haindl, Sonja Hochmeister

**Affiliations:** Department of General Neurology, University Clinic of Neurology, Medical University of Graz, Graz, Austria

**Keywords:** multiple sclerosis, vitamin D, animal models-rodent, autoimmune diseases, therapy

## Abstract

Multiple sclerosis is a multifactorial disease of the central nervous system with both genetic and environmental causes. The exact disease mechanisms are still unclear. Consequently, studies of possible treatment and preventive measures cover a large setting of heterogeneous approaches. Vitamin D is one of these approaches, and in many trials the relation of vitamin D serum levels and multiple sclerosis disease risk and activity describes different effects with sometimes inconsistent findings. Animal models are substantial for the research of disease mechanisms, and many of the drugs that are currently in use in multiple sclerosis have been developed, tested, or validated *via* animal studies. Especially when clinical studies show contradicting findings, the use of standardized settings and information about the mechanistic background is necessary. For this purpose, animal models are an essential tool. There is a variety of different experimental settings and types of animal models available, each of them with own strengths but also weaknesses. This mini-review aims to overview results of vitamin D studies in different animal models and sums up the most important recent findings.

## Introduction

**Figure 1:**
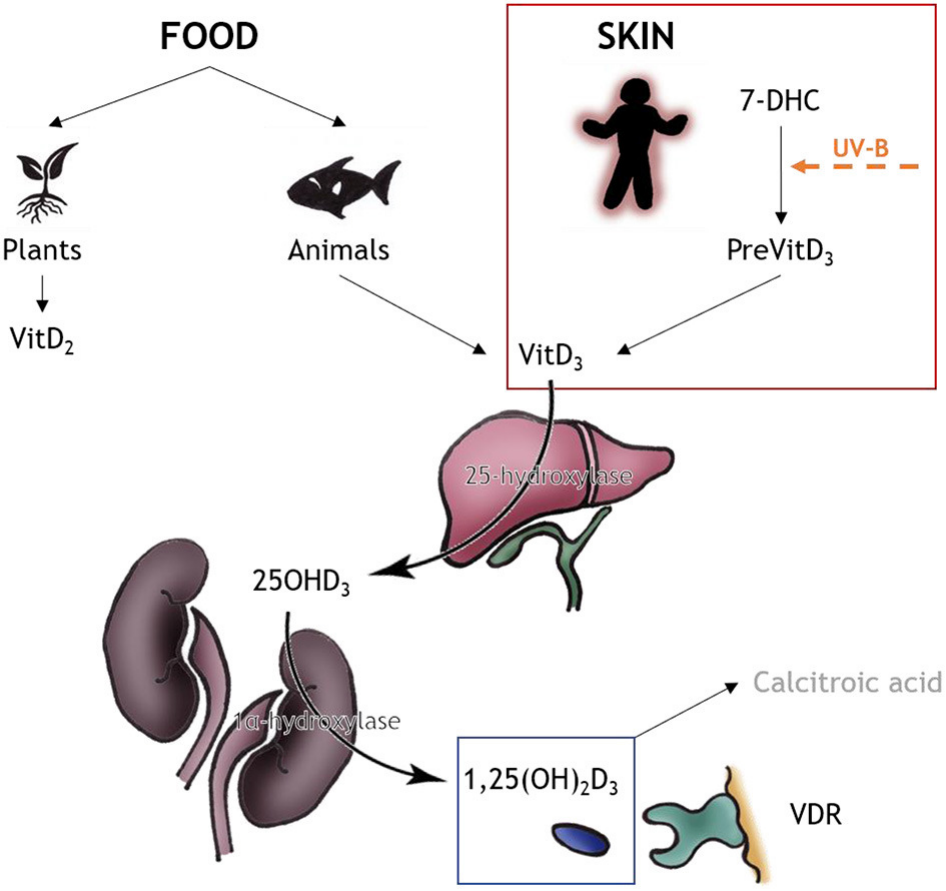
VitD metabolism. VitD can be obtained from two sources, food (minor part) and skin (major part). The source for vitD_2_ are plants and fungi, and the source of vitD_3_ are animals, especially fatty fish. The major source of vitD_3_ is produced nonenzymatically *via* the human skin: 7-DHC converts to PreVitD which isomerizes *via* heat to vitD_3_. In the liver, vitD_3_ is converted to 25OHD_3_ *via* 25-hydroxylase; in the kidneys the biologically active form 1,25(OH)_2_D_3_ is produced *via* 1alpha-hydroxylase. The active product either can bind to VDR for correspondent outcome or is converted to calcitroic acid for storage. Information upon vitD metabolism obtained from (8); drawings by MT Haindl.

Multiple sclerosis (MS) is a chronic demyelinating disease of the central nervous system (CNS), affecting about 2.5 million people worldwide. It is an autoimmune disease with targeted myelin attack that causes demyelination (1, 2). Even though disease-modifying medications are capable to reduce disease severity, the disease continues to worsen over the patient's life span. Both genetic and environmental factors contribute to disease development, but the exact mechanisms are still not fully understood. Experimental autoimmune encephalomyelitis (EAE) in rodents is the favored model for exploring neuroinflammatory aspects of the disease, while toxin-induced demyelinating models like the cuprizone model are able to elucidate the cellular mechanism of de- and remyelination (2–5). Vitamin D (vitD), or the lack of it, is one frequently discussed environmental factor associated with MS, and its immunomodulatory ability has been widely demonstrated (6, 7). Despite numerous studies suggesting a beneficial effect of vitD intake in MS, there is still a controversy whether the supplementation can be used therapeutically (7). This work will discuss and summarize recent data from animal models on this topic. For overview, in Table 1 and Figure 1 the chemical and metabolic background of the vitD metabolism is summed up.

**Table 1:** Chemical and metabolic background of vitD.

**Shortcut**	**Explanation**
vitD	In this manuscript the shortcut vitD sums up vitaminD_3_ and any intermediate of vitaminD_3_
vitaminD_3_	Cholecalciferol (inactive)
vitaminD_2_	Ergocalciferol
7-DHC	7-Dehydrocholesterol
25OHD_3_	25-HydroxyvitaminD_3_
1,25(OH)_2_D_3_	1,25-DihydroxyvitaminD_3_, calcitriol (active)
VDR	vitD receptors

*There are different chemical forms of vitD to be distinguished (in this work, the shortcut vitD sums up vitaminD3 and any intermediates). There are two sources of vitD; the majority is generated via the skin in a non-enzymatic process; the minor part is gained via food. The starting product 7-DHC is converted to Pre-vitD_3_ via UV-B irradiation. This pre-vitamin isomerizes to vitD_3_ in a thermo-sensible process. VitD_3_ is converted to 25OHD_3_ in the liver. The biologically active form of this vitamin is 1,25 (OH)_2_D_3_, generated via hydroxylases in the kidneys. This active form is able to bind to VDRs, transcription factors, present in nearly every tissue. Alternatively, 1,25 (OH)_2_D_3_ can be converted to the biologically inactive form calcitroic acid for storage (1, 8)*.

## Disease Prevention and Risk Reduction

Most studies investigating the capability of vitD to prevent MS are based on EAE findings. There is prophylactic potential of the association of myelin oligodendrocyte glycoprotein (MOG) peptide and active vitD against EAE. Vaccination with a mixture of MOG associated with vitD determines a reduction in CNS inflammation, dendritic cell maturation, clinical score, body weight loss, and production of cytokines, indicating that this association tones down the autoimmune response and prevents EAE. In other autoimmune conditions, there is a similar effect expectable (9). However, not only immunization with a MOG-vitD mixture has preventive effects. Also, very early intervention with the active form of vitD controls neuroinflammation during EAE development and was shown to decrease prevalence, clinical score, inflammation, and demyelination. Furthermore, a reduced major histocompatibility complex class II (MHCII) expression in macrophages and microglia as well as the level of oxidative stress and messenger RNA (mRNA) expression for caspase-1, interleukin (IL)-1β, and others was observed. These effects are accompanied by stabilization of blood–spinal cord barrier permeability, indicating that early intervention with vitD can control the neuroinflammatory process which is one of the hallmarks of EAE and MS (6). Conversely, low vitD levels are associated with increased risk of MS, suggesting the possibility of a gene–environment interaction in MS pathogenesis. VitD supplementation downregulates the MHCII expression in EAE. The impact of vitD on one master regulator of MHCII expression was investigated in 2020 on EAE rats. An inverse vitD dose-dependent effect on demyelination and inflammatory infiltration of the CNS, as well as downregulation of some pro-inflammatory genes, indicated an impact of vitD on pathophysiology and immune response during EAE. A modulatory effect of vitD regarding genetic variances in MS is therefore most likely probably relevant for the human disease as well (10). Additionally, vitD may reduce the MS risk in part through a mechanism involving myeloid cell vitD production and CTLA-4 upregulation in CNS-infiltrating T-cells. Humans with CTLA-4-inactivating mutations have an incompletely penetrant cellular phenotype with hyperactive effector CD4+ T-cells and a complex immune dysregulation syndrome. Another EAE study found out that CTLA-4 might act as a vitD-regulated immunological checkpoint in MS prevention (11).

## Influence on T-Cells

The protective effect of vitD associates with decreased proliferation of CD4+ T-cells and a lower frequency of pathogenic T-helper (Th) 17 cells. Multiple pathways, critical for T-cell activation and differentiation, seem to be affected by vitD. For example, Jak/Stat, Erk/Mapk, and pi3K/Akt/mTor signaling pathway genes were downregulated upon vitD supplementation. VitD might modulate MS risk by changing myelin-reactive T-cell expression patterns as observed in EAE. Additionally, the role of vitD supplementation for prevention or treatment of autoimmune diseases in general is supported because CD4+ T-cells are driving target organ destruction in autoimmune diseases and many of the autoimmune loci are shared by multiple autoimmune diseases (7). However, the influence of vitD on T-cells seems to act not only *via* metabolic pathways but also upon dendritic cells (DCs). DCs mediate immune response *via* their antigen presentation function, driving T-cell differentiation. VitD has the ability to induce tolerogenic DCs (VD3-DCs), increasing the negative regulatory signaling pathway programmed death 1 (PD1)/programmed death ligand 1 (PDL1). The expression of PD1 and PDL1 increases significantly after vitD treatment, enhancing the activation of this pathway. As a result, the activation of T-cells can be inhibited and the number of Tregs is increased, promoting immune tolerance (12). The induction of VD3-DCs further inhibits the infiltration of T helper type 1 (Th1) and Th17 cells into the spinal cord and increases the proportions of regulatory T-cells and regulatory B-cells in peripheral immune organs, thereby attenuating EAE (13). One dose of calcitriol plus vitD is able to reverse EAE, resulting in increased CD4+ T-cell transcripts, Helios protein, CD4+ Helios+ FoxP3+ Treg, and global DNA methylation. Calcitriol might drive a transition from CD4+ T-cell to Treg cell dominance, recycling homocysteine to methionine, reducing homocysteine toxicity, maintaining DNA methylation, and stabilizing CD4+ Helios+ FoxP3+ Treg. Structural similarity in the responsible vitD-promoters even suggests a similar regulatory mechanism for humans (14). CD4+ T-cells have a cooperative amplification loop promoting CD4+Helios+FoxP3+ Treg development, and this process is disturbed when the vitD pathway is impaired (15).

Because of the role of T-cells in MS, glucocorticoids remain the most commonly used substance in treating acute MS relapses. However, in approximately 30% of patients, a limited efficacy of glucocorticoids is reported, often in patients with low serum vitD levels. VitD increases glucocorticoid-induced apoptosis of T-cells *via* upregulation of the glucocorticoid receptor (GCR). With the help of two different EAE models with reduced or absent GCR signaling, it was demonstrated that there are synergistic effects of vitD and glucocorticoids, probably mediated through mTORc1 signaling. Severe vitD deficiency is associated with downregulation of an mTORc1 inhibitor in human T-cells. In animals with T-cell-specific depletion of mTORc1 and in animals receiving a specific mTORc1 inhibitor, the synergistic effects of vitD/glucocorticoids on GCR upregulation, T-cell apoptosis, and therapeutic efficacy in EAE failed (16).

Beside the direct influence of vitD on T-cells, also related molecules such as cytokines and chemokines can induce powerful changes. For example vitD increases the production of IL-4, IL-10, and TGF-β while decreasing IFNγ, IL-6, TNFα, and IL-17 production accompanied with a deviated balance between Th1/Th2 and Th17/Treg to Th2 and Treg under middle and high doses of vitD (17). Accordingly, vitD downregulates the expression of some Th17 cell-related cytokines, key inflammatory chemokines, and chemokine receptors in EAE considering a possible therapeutic potential of vitD in future treating MS (18).

## Remyelination

There is also recent literature available describing the effects of vitD on remyelination. Most data are available on the cuprizone model, since it is the easiest way of studying de- and remyelination. One study found that there is a significant increase MOG and 2′,3′-cyclic-nucleotide 3′-phosphodiesterase (CNPase) expression in vitD-supplemented cuprizone-exposed mice compared to control groups. MOG is a minor component of the myelin sheath, but it has an important autoantigen link to the pathogenesis of EAE whereas the protein CNPase is one of the main proteins of myelin and its appearance seems to be one of the earliest events of oligodendrocyte differentiation and myelination. VitD may play a role in the process of remyelination by increasing MOG and CNPase expression in the cortex (19). In another study, axonal damage during de- and remyelination in the cuprizone mouse model was investigated. The authors found significantly higher neurofilament preservation in the high dose-supplemented inactive vitD group in comparison to the low dose-supplemented group. High doses of active vitD, however, given after the demyelination phase as well as during remyelination did not influence axonal regeneration, while inactive vitD, given before and during cuprizone exposure, seems to have a protective effect on axons (20). VitD might even have the ability to trigger neuronal stem cell differentiation (21).

## VitD and MS Progression

After a disease duration of about 20 years, most MS patients enter the progressive state of the disease with a steady worsening of clinical neurological symptoms. Only little data are available upon the question whether vitD could be a reasonable support during progressive MS. Some clinical studies suggest a protective role of higher vitD levels on myelin content in progressive MS and an association between a low vitD status at the beginning of MS and the early entry to the progressive disease state (22, 23). A most recent study however could not confirm these assumption—vitD levels were not associated with the severity of optical coherence tomography findings or low-contrast letter acuity in their group of progressive MS patients (24). Clinical studies may furthermore be hampered by the possibility that severely affected progressive MS patients may have limited sunlight exposure as a consequence of their disease rather than as a cause. This demonstrates the need of more mechanistic knowledge of the mode of action of vitD in progressive MS. Unfortunately, there is no animal study addressing this research question so far. More studies making use of recently established animal models of progressive MS would be most welcome to elucidate the mechanistical background of how vitD could affect this disease state (25, 26).

## Issues and Problems

### VitD Controversy

Even though the majority of animal studies affirm a beneficial effect of vitD in experimental animal models of MS, there is also a small list of literature suggesting that vitD is not capable of positively influencing autoimmune diseases. VitD and sunlight have each been reported to protect against the development of EAE. Since exposure of ultraviolet (UV) light also causes the generation of vitD, studies investigated whether the UV-based suppression of EAE results, at least in part, from the production of vitD. One study examined UV suppression of EAE in mice devoid of vitD receptor (VDR) and mice unable to produce 7-DHC. UV light suppression of EAE occurred in the absence of vitD production and in the absence of VDR (27). However, it is possible that the UV suppression of EAE can further be influenced by the active form of vitD. The presence of active vitD surprisingly actually counteracted the suppressive effect of UV in one study (28). Further investigations should focus on identifying the pathway responsible for the protective action of UV in EAE and presumably human MS (27). Two independent research groups have demonstrated unexpectedly that vitD deficiency blocks EAE development. In one study, the suppression of EAE is even reported as a result from hypercalcemia and not as an effect of the active form of vitD (28). Another study revealed that a NBUVB light at 311 nm is responsible for the EAE suppression, and this wavelength does not produce vitD. There are suggestions upon a mechanism of EAE suppression independent of vitD, whereas a remaining question is still whether the active form of vitD has any impact on the NBUVB suppression of EAE (28, 29). These findings emphasize the need of further mechanistic research to gain a better understanding of EAE suppression and the role of vitD and light.

### VitD Supplementation: Attention Should Be Paid to Adequate Dosage

The problem of potential overdosing vitD resulting in hypercalcemia is a critical aspect to this topic. Moderate supplementation of vitD reduces the severity of subsequent EAE in mice, associated with an expansion of Tregs. Direct exposure of T-cells to vitD metabolites inhibits their activation. On the other hand, high doses of vitD (200 nmol/l) in mice result in fulminant EAE with massive CNS infiltration. This is caused by mild hypercalcemia only observed in animals receiving high, but not medium, doses of vitD (30). Because of this problem, one study investigated the therapeutic potential of Paricalcitol (Pari) on EAE, since it is a non-hypercalcemic vitD_2_ analogue, capable of promoting anti-inflammatory activity in kidney and heart diseases. In this study, severity, apoptosis and neuropathology of EAE were reduced *via* Pari accompanied by inhibition of glial cell activation, cellular infiltration, pro-inflammatory molecules, and activation of nuclear factor κB (NF-κB). This phenomenon could further be reduced by suppressing NF-κB with its inhibitor and Pari in combination (31). On the contrary, another study found a lower production of proinflammatory cytokines and reduced inflammation only in the EAE/vitD group, not in the Pari group. The authors thus suggest, that vitD, but not Pari, has the potential to be used as a preventive therapy to control MS severity (32). Another approach to bypass the problem of vitD overdose suggests a combination of vitamin A (vitA) and vitD. The combinatory treatment with vitA and vitD using the optimal synergistic effects with low doses could be beneficial in addressing the side effects and possibly paving the way for a more efficient MS therapy. One study demonstrated a significant different cytokine gene expression profile in the treated and control groups, suggesting a benefit of this treatment approach (33).

### Experimental Animal Models and Problems in Translation

Results from animal models have to be critically validated. Common EAE models reflect important aspects of MS, but one has to consider that these models are mainly based on inflammation induced by autoreactive CD4+ T-cells whereas results from clinical trials in MS indicate that CD8+ T-cells and B-lymphocytes may play an important role in MS. In EAE, the inflammatory demyelinating disease burns out when the peripheral brain antigen depot has been removed. Therefore, it is most likely that in human MS, a persistent trigger within or outside the CNS is required for chronic disease propagation (4). This emphasizes the need of a variety of carefully selected animal models to cover different aspects of different phases of MS. However, especially concerning progressive MS, data are currently scarce.

### VitD and Clinical Data in MS

Most of the clinical data regarding vitD and MS focus on its ability to reduce the risk of MS development. The suggestion that low vitD serum level is one MS risk factor is nowadays mainly accepted. One important question however remains upon its actions once the disease has started. Many studies concerning with this question are unfortunately insufficiently powered, most often without a long-lasting follow-up or with methodological bias, hindering conclusive results. Nevertheless, it appears highly likely that vitD is able to decrease components of the inflammatory pathway of the disease. Of course, further scientific validation is needed; a systematic vitD supplementation of MS patients has already been recommended in clinical practice, anyway (34). In comparison to the evidence of benefit of vitD supplementation in early MS, there is little known about the role of vitD in the progressive disease phase. Even though it is well known that these patients commonly suffer from low vitD serum levels, there is still the requirement of long-term observational studies (23, 35).

## Discussion

**Figure 2:**
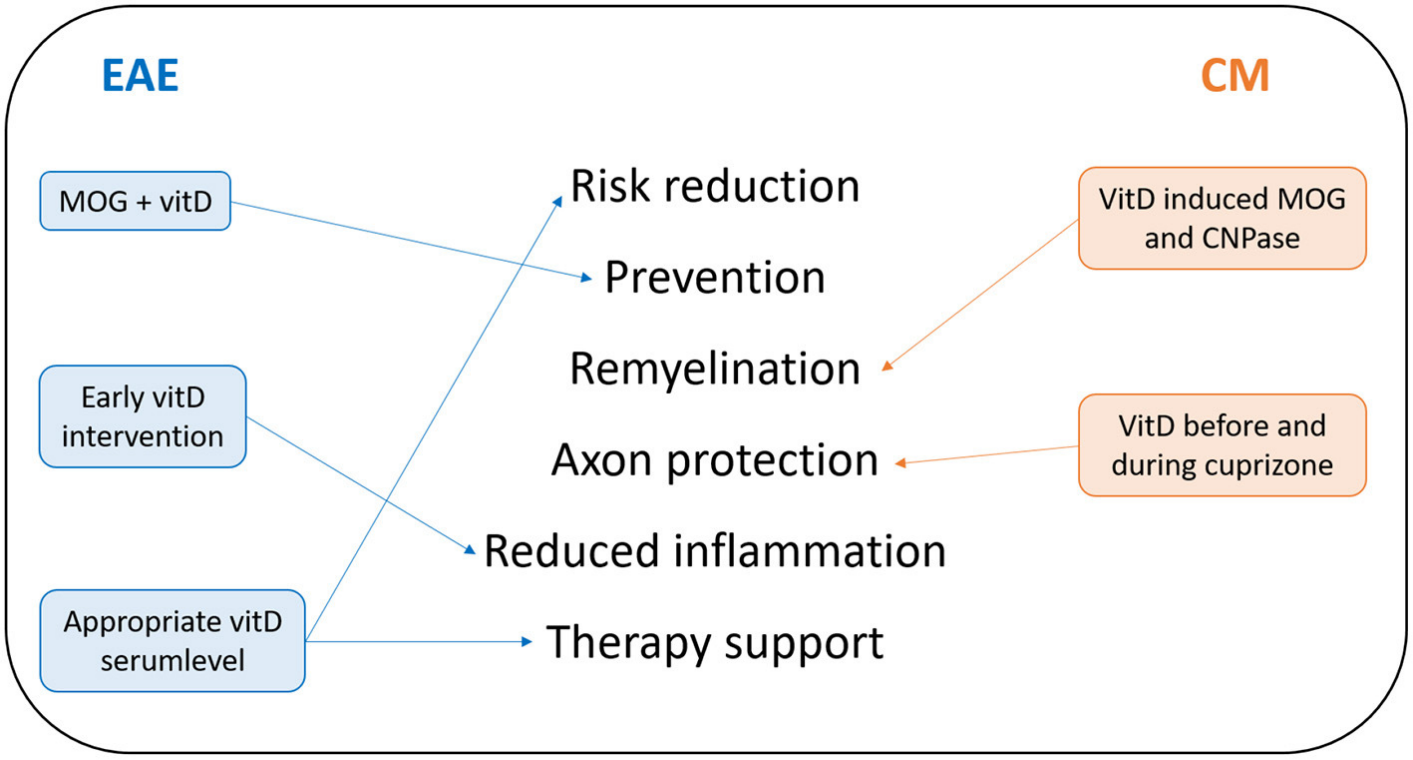
Summary of recent findings of protective vitD effects. Most results upon vitD effects in MS-related animal models were obtained by EAE and the cuprizone model (CM). Lessons from EAE are written on the left side in blue; lessons from CM are shown in orange on the right side. In the middle of this summary, recent research findings of protective effects of vitD are listed.

Numerous animal studies attest benefits of vitD. Based on the current state of knowledge, vitD supplementation may be considered as a preventative measure for decreasing the risk for developing autoimmune diseases and potentially as adjunctive therapy (7). Figure 2 sums up the most recent findings discussed in this work. Studies on the beneficial effect of vitD in EAE suggest that treatment with vitD before EAE induction or from peak disease is effective at reducing disease severity. This beneficial effect may be mediated at least in part through the attenuation of T-cells, reduction of axonal and neuronal loss, and support of oligodendrocyte maturation (36, 37). Some other recent studies focused on genetic variations and how vitD could intervene. So far, it has been shown that vitD promotes negative feedback regulation of Toll-like receptor (TLR) signaling in macrophages *in vitro*, which in turn ameliorates inflammation. Naturally occurring allelic differences in the Vra4locus/Mhc2ta could be relevant for the efficacy of vitD in modulation of MS-like neuroinflammation and potentially even in MS, but sufficient information about response to vitD within the Vra4 gene locus is still missing (10). Data from EAE allow the conclusion that vitD synthesis by activated microglial cells and macrophages in the CNS preserves neurological function by dampening the inflammatory process. VitD seems to support the CTLA-4 immunological checkpoint to prevent immune-mediated neurological damage. Additionally, the protective effect of vitD seems to involve epigenetic mechanisms (DNA methylation), which may provide a molecular basis for cellular memory that mediates long-term effects and suggests potential for future combined therapies (7). VitD and VDR are closely associated with the development of autoimmune diseases. There might be unknown factors capable of regulating VDR. It is already known that miRNAs are associated with VDRs. Even though the involvement of miRNAs in human diseases strengthened our understanding of pathogenesis, candidates for miRNAs that have the potential to control autoimmune pathomechanisms remain limited (38). Those strong regulatory molecules could become powerful tools of autoimmune disease management, if the exact mechanisms are elucidated in further studies. Beside validated findings on vitD beneficial effects, one important aspect is the reasonable application of vitD. Findings of studies regarding dose dependence of vitD suggest that vitD at moderate levels may exert a direct regulatory effect, while continuous high-dose vitD treatment could trigger MS disease activity by raising T-cell excitatory calcium (30, 39). This is indeed a very important lesson from animal studies since hypercalcemia was reported also in humans supplemented with high doses of vitD (40, 41). Monitoring the vitD level in MS patients thus is crucial to ensure positive effects of the supplementation. Another important topic is the impact of vitD on different MS treatments. Current MS treatments are found to be directly or indirectly linked to NF-κB pathways and act to adjust the immune system. MS is associated with constitutive activation of NF-κB, which results in excessive expression of related effector molecules, driving inflammation, and there is a very complex association of this factor and different cytokine patterns involved in EAE progression too (31). Pari could be one potential NF-κB blocker, and other vitD analogues might act in a similar way. Again, most of our knowledge about NF-κB is based on results from animal studies and further animal studies will be needed to investigate the mechanism further (31, 32). A similar issue concerns MS therapies working by increasing PDL1 expression. The PD1/PDL1 pathway might act as a key player in the mechanism of demyelination and autoimmune response due to its regulation of antigen-presenting cell and T-cell interaction. VD3-DCs attenuate the clinical symptoms of EAE by increasing the activation of the PD/PDL1 signaling pathway. However, the specific mechanisms of this signaling pathway still remain unsolved and further research is necessary in order to apply VD3-DCs to clinical practice (12). Translation of data from preclinical models to humans is always to be used with caution. However, it is still necessary to investigate pathophysiological mechanisms with the help of animal models. Even though most preclinical assays indicated a strong potential of vitD as a useful agent for EAE prevention or therapy, clinical trials with patients revealed mixed data (32). One possible reason is the missing knowledge in how vitD exactly acts in many different signaling pathways. Another interesting research goal is to elucidate gender aspects of vitD effects (42, 43). In general, autoimmune diseases are characterized by a significant female bias. This is also the case in MS where more females are affected (43). This sexual dimorphism in autoimmune diseases seems to be related to sex hormones, which differently affect the immune system. In general, males show higher immunosuppression may be due to androgens, and females show a higher immunoreactivity and competence likely related to estrogens. This leads to a greater resilience to infections but also to a higher risk for developing autoimmune diseases (42, 43). Additionally, the outcome of vitD status in MS is determined by gene-by-sex interactions (44). Thus, gender and sex hormones could be included as variables when evaluating the potential power of vitD to influence autoimmune diseases (42, 43). Beside all these findings, one has to consider that there is a developmental stage-dependent efficiency of vitD to ameliorate neuroinflammation, suggesting that childhood and adolescence should be the target for the most effective preventive vitD treatment (45).

## Conclusion

The majority of literature suggests a beneficial role of vitD at least in therapy of MS related animal models. When it comes to the translation of these findings to the human situation, the most important aspect to be considered is the right dosage, to avoid negative side effects. Nevertheless, for an effective treatment or support of MS therapies with the help of vitD and probably other vitamins, further studies are necessary. Especially if and how exactly vitD could intervene in pathophysiological mechanisms of progressive MS remains largely unsolved.

## Author Contributions

MH wrote the original draft and generated the figures. All authors contributed to the writing of this article, approved the submitted version, were involved in developing the plan for the article, and in reviewing and editing the manuscript.

## Conflict of Interest

The authors declare that the research was conducted in the absence of any commercial or financial relationships that could be construed as a potential conflict of interest.

## Publisher's Note

All claims expressed in this article are solely those of the authors and do not necessarily represent those of their affiliated organizations, or those of the publisher, the editors and the reviewers. Any product that may be evaluated in this article, or claim that may be made by its manufacturer, is not guaranteed or endorsed by the publisher.
